# Cost-effectiveness analysis of lemborexant for treating insomnia in Japan: a model-based projection, incorporating the risk of falls, motor vehicle collisions, and workplace accidents

**DOI:** 10.1017/S0033291722000356

**Published:** 2022-10

**Authors:** Shunya Ikeda, Mie Kasai Azuma, Kenichi Fujimoto, Hidetoshi Shibahara, Sachie Inoue, Margaret Moline, Mika Ishii, Kazuo Mishima

**Affiliations:** 1 Department of Public Health, School of Medicine, International University of Health and Welfare, Narita, Japan; 2 Eisai Co., Ltd., Tokyo, Japan; 3 CRECON Medical Assessment Inc., Tokyo, Japan; 4 Graduate School of Health and Welfare, International University of Health and Welfare, Tokyo, Japan; 5 Eisai Inc., Woodcliff Lake, NJ, USA; 6 Department of Neuropsychiatry, Akita University Graduate School of Medicine, Akita, Japan

**Keywords:** Cost-effectiveness, insomnia treatment, lemborexant, QALY, suvorexant, zolpidem

## Abstract

**Background:**

Lemborexant has demonstrated statistically significant improvements in sleep onset and sleep maintenance compared with placebo and zolpidem tartrate extended release, measured both objectively using polysomnography and subjectively using sleep diaries, in the phase 3 clinical trial SUNRISE 1. This study evaluated the cost-effectiveness of lemborexant compared with suvorexant, zolpidem immediate release (IR), and untreated insomnia.

**Methods:**

A decision-tree model was developed for falls, motor vehicle collisions, and workplace accidents associated with insomnia and insomnia treatments from a Japanese healthcare perspective and with a 6-month time horizon. The model extracted subjective sleep onset latency treatment responses and disutility values for non-responders from SUNRISE 1. Cost-effectiveness was assessed using incremental cost per quality-adjusted life year (QALY) gained. One-way and probabilistic sensitivity analyses were conducted to evaluate the impact of parameter uncertainty on the results.

**Results:**

In the base-case analysis, the mean estimated QALYs for lemborexant, suvorexant, zolpidem-IR, and untreated insomnia were 0.4220, 0.4204, 0.4113, and 0.4163, and expected medical costs were JPY 34 034, JPY 38 371, JPY 38 139, and JPY 15 383, respectively. Lemborexant saved JPY 4337 and JPY 4105 compared with suvorexant or zolpidem-IR, respectively, while conferring QALY benefits. The incremental cost-effectiveness ratio (ICER) of lemborexant compared with that of untreated insomnia was JPY 3 220 975 /QALY. Lemborexant was dominant over suvorexant and zolpidem-IR and was cost-effective when compared with untreated insomnia. Sensitivity analyses supported the results' robustness.

**Conclusions:**

In a Japanese clinical practice setting, lemborexant may represent a better investment for treating insomnia in the healthcare system in Japan.

## Introduction

Insomnia is a common, patient-reported complaint that is characterized by difficulty falling asleep or maintaining sleep, premature wakening, or a combination of both sleep onset and sleep maintenance issues (Sateia, Sherril, Winter-Rosenberg, & Heald, 2017b). Insomnia is more common in women than in men (Zhang & Wing, 2006), and at lower levels of education (Gellis et al., 2005). Untreated insomnia causes many difficulties for patients in terms of increased fatigue, reduced quality of life (QOL), impaired daytime functioning, and an increased risk of accidents and injuries (Chen, Lee, & Buxton, 2017; Lombardi, Folkard, Willett, & Smith, 2010; Olfson, Wal, Liu, Morin, & Blanco, 2018). Although cognitive behavioral therapy is standard treatment for the management of patients with insomnia, pharmacotherapy is an important treatment option for many patients, with hypnotics being the most commonly prescribed medication (Sateia, Buysse, Krystal, Neubauer, & Heald, 2017a).

The hypnotic drugs approved for the treatment of insomnia in Japan include benzodiazepines (BZDs), non-BZDs, melatonin receptor agonists, and dual orexin receptor antagonists (DORAs). Although BZDs and non-BZDs are widely used, these drugs are associated with a risk of physical dependency and with an increased risk of falls, motor vehicle collisions (MVCs), problems with memory, and daytime sedation, especially in older persons (Mishima, 2015; Uchiyama, 2019; Wang, Bohn, Glynn, Mogun, & Avorn, 2001). As such, BZDs are not recommended for use in the elderly in Japan (Mishima, 2015; Pharmaceuticals and Medical Devices Agency, 2017; Uchiyama, 2019). Furthermore, although melatonin receptor agonists may present a lower risk of harm in the elderly (Uchiyama, 2019), they are only approved for improving sleep onset, not for sleep maintenance.

DORAs are a new class of drug developed to address the limitations of the previously approved drug classes (Jacobson, Chen, Mir, & Hoyer, 2017) and are effective for improving sleep maintenance, an advantage that has not been established for BZDs, except for long-acting BZDs, and most non-BZDs (Herring et al., 2012; Herring et al., 2016; Sateia et al., 2017a; Uchiyama, 2019). In 2014, suvorexant became the first DORA to be approved in Japan, although it is only recommended for improving sleep maintenance (Sateia et al., 2017a; Uchiyama, 2019). This recommendation is based on studies of suvorexant in elderly patients that showed clinically meaningful improvements in sleep maintenance [wake after sleep onset (WASO)], but not sleep onset [latency to persistent sleep (LPS)] (Herring et al., 2012, 2016).

Lemborexant is a new DORA that was approved in Japan in January 2020 following approval in the United States; it was also recently approved in Canada. In the SUNRISE 1 trial (NCT 02783729; E2006-G000-304), lemborexant demonstrated superior efficacy over zolpidem tartrate extended release (ER) and placebo in older adults (Rosenberg et al., 2019). After one month, treatment with lemborexant 5 and 10 mg resulted in clinically meaningful improvements over placebo in terms of LPS and WASO measures. During the first seven nights of treatment and at the end of the month, the mean decreases from baseline in terms of log-transformed subjective sleep onset latency (sSOL) were also significantly greater for lemborexant 5 and 10 mg compared with those of zolpidem ER 6.5 mg or placebo (Rosenberg et al., 2019). Findings from the SUNRISE 2 trial (NCT02952820; E2006-G000-303), which was conducted over periods of 6 (Kärppä et al., 2020) and 12 months (Yardley et al., 2020) in adults aged ⩾18 years, showed that the improvements in subjective sleep onset and sleep maintenance induced by lemborexant were sustained in the long term and that lemborexant was well-tolerated.

Several studies have examined the impact of insomnia on healthcare costs and utilization (Gamaldo et al., 2016; Wickwire et al., 2020). However, information on the cost-effectiveness of drug treatments among elderly individuals with insomnia is limited (Mohit & Cohen, 2019; Tannenbaum et al., 2015; Wickwire et al., 2020). The present study was designed to assess the cost-effectiveness of treatment with lemborexant compared with that of suvorexant or zolpidem immediate release (IR), which is commonly used in Japan, as well as untreated insomnia, with a particular focus on the impact of treatment on falls, MVCs, and workplace accidents (WPAs).

## Methods

### Model structure and framework of the economic analysis

**Fig. 1.**
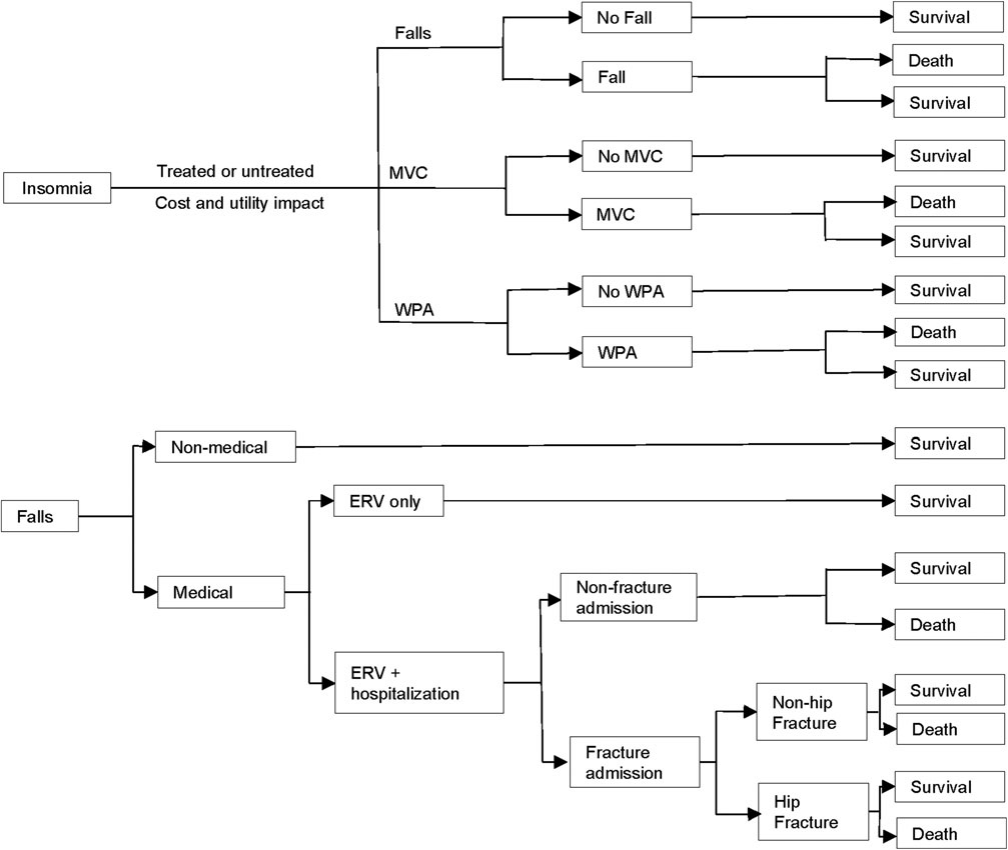
Flow diagram of the model structure. ERV, emergency room visits; MVC, motor vehicle collisions; WPA, workplace accidents.

A decision-tree model was developed to project the costs and health outcomes of lemborexant, suvorexant, zolpidem IR, and of untreated insomnia in terms of the following three events associated with insomnia and its treatments: falls, MVCs, and WPAs (Fig. 1). Each of these events incurred a cost, a decrement in utility, and a probability of death. Patients in each treatment group incurred costs associated with medical care for chronic insomnia, including drug prescriptions, and a utility decrement with residual chronic insomnia. Although treatment efficacy was not considered to be age-dependent, the evaluation model was applied to younger adults (18–64 years) and older adults (⩾65 years) to take into account age-related differences in the incidence of falls, MVCs, and WPAs in the general population (MHLW, 2019; Niino, Tsuzuku, Ando, & Shimokata, 2000; NPA, 2019).

The cost-effectiveness analysis was conducted in accordance with the Japanese Ministry of Health, Labour and Welfare (MHLW) guidelines (C2H, 2019) from the Japanese healthcare perspective typically adopted by payers. The health outcomes of each intervention were evaluated in QALYs, and the analysis only included direct medical costs. Although long-term administration of insomnia medication is not recommended (Mishima, 2015; Sateia et al., 2017a), many patients use insomnia medications for long periods in real-world clinical practice (Katz & McHorney, 1998), and an analysis of their long-term use is highly justified. Therefore, the time horizon was set at six months, and no discount rate was applied for less than one year. Cost-effectiveness was assessed using incremental cost-effectiveness ratios (ICER) and a willingness-to-pay (WTP) threshold of JPY 5 million per QALY gained (Hasegawa, Komoto, Shiroiwa, & Fukuda, 2020). Unit costs were based on the 2020 Japanese fee schedule and drug tariffs, each of which was defined by the MHLW at an exchange rate of USD 1 = JPY 107 (September 2020) (Bank of Japan, 2020). All analyses were performed using Microsoft Excel.

### Treatment efficacy

The efficacy of lemborexant in this study was based on that in the SUNRISE 1 trial, a global, randomized, double-blind, parallel-group, placebo-controlled, active-comparator phase 3 study (Rosenberg et al., 2019). The primary endpoint was the change in LPS from baseline for lemborexant compared with that of placebo, and the secondary endpoints included WASO and sSOL changes. The current analysis was based on the sSOL response rate because the use of subjective, rather than objective, measures are recommended by the Japanese regulatory authority (Pharmaceuticals & Medical Devices Agency, 2011). A response was defined as an sSOL ⩽20 min, provided the mean baseline sSOL was >30 min.

The response rates for suvorexant and zolpidem IR were based on a network meta-analysis (NMA) aimed at providing estimates of comparative efficacy and acceptability for up to 16 pharmacological treatments, including lemborexant, for insomnia disorder in adults (McElroy et al., 2021). The response rate for zolpidem IR was assumed to be the same as that for zolpidem ER, as the mean decrease in sSOL from baseline at four weeks did not significantly differ [−8.3 min; 95% credible interval (Crl), −17.5 to 0.4] between zolpidem IR and zolpidem ER (McElroy et al., 2021), and a direct comparison of responder rates for zolpidem ER in SUNRISE 1 was available. The response rate for suvorexant was conservatively assumed to be the same as that for lemborexant, as the data describing responder rates were unavailable due to the fact that no direct comparison was performed in SUNRISE 1. However, the NMA found that the difference in the mean decreases in the sSOL values from baseline at three months for lemborexant compared with that of suvorexant was −7.1 min (95% Crl; −13.2 to −0.9), a difference that was statistically significant (McElroy et al., 2021).

Based on the findings from SUNRISE 2 (Kärppä et al., 2020; Yardley et al., 2020), the present model assumed that the efficacy of each treatment was maintained for the duration of the model's time horizon. Using the subgroup analyses from SUNRISE 1 and SUNRISE 2, which showed that treatment effects did not vary with age, it was assumed that treatment efficacy was not age-dependent. The response rate for untreated insomnia was set 0, corresponding with no improvement in insomnia symptoms.

### Events

The baseline risks and the risks associated with insomnia or insomnia treatment for each event within the model were estimated from previously published reports and government data (Table 1).

**Table 1.** Model inputs

		Value	1-way SA Setting			PSA Setting		
Parameter			Lower	Upper	Range	s.e.	Distribution	Source
*Clinical inputs*								
Responder rate (proportion experiencing improved sleep)								
sSOL	Lemborexant	0.207	0.168	0.247	95%CI	0.020	Beta	Rosenberg et al. (2019)
	Suvorexant	0.207	0.168	0.247	95%CI	0.020	Beta	Same as lemborexant
	Zolpidem	0.113	0.070	0.157	95%CI	0.022	Beta	Same as zolpidem ER in SUNRISE 1 (Rosenberg et al., 2019)
	Untreated insomnia	0	–	–	–	–	–	Assumption
WASO	Lemborexant	0.455	–	–	–	–	–	Rosenberg et al. (2019)
	Suvorexant	0.455	–	–	–	–	–	Same as lemborexant
	Zolpidem	0.349	–	–	–	–	–	Same as zolpidem ER in SUNRISE 1 (Rosenberg et al., 2019)
	Untreated insomnia	0	–	–	–	–	–	Assumption
LPS	Lemborexant	0.377	–	–	–	–	–	Rosenberg et al. (2019)
	Suvorexant	0.377	–	–	–	–	–	Same as lemborexant
	Zolpidem	0.214	–	–	–	–	–	Same as zolpidem ER in SUNRISE 1 (Rosenberg et al., 2019)
	Untreated insomnia	0	–	–	–	–	–	Assumption
Proportion of patients	Younger	51.2%	–	–	–	–	–	Ministry of Health, Labour and Welfare (2019)
	Older	48.8%	–	–	–	–	–	
Mean age	Younger	48.7	–	–	–	–	–	
	Older	74.7	–	–	–	–	–	
Falls								
Hazard ratio	Lemborexant	1	0.800	1.200	± 20%	0.100	Log-normal	McElroy et al. (2021)
	Suvorexant	1.26	1.008	1.512	± 20%	0.126	Log-normal	
	Zolpidem	2.4	0.92	6.27	95%CI	0.49	Log-normal	Treves et al. (2018)
	Untreated insomnia	1.48	1.184	1.776	± 20%	0.148	Log-normal	Stone et al. (2008)
Annual probability of a first fall	Younger	12.9%	10.0%	15.8%	95%CI	1.47%	Beta	Niino et al. (2000)
	Older	16.5%	13.3%	19.7%	95%CI	1.64%	Beta	Niino et al. (2000)
Mean extra falls/faller in general population		0.957	0	1.02	95%CI	0.286	Gamma	U.S. Centers for Medicare & Medicaid Services (2019)
Slope of extra falls with proportion of first falls		2.5	–	–	–	0.26	Normal	U.S. Centers for Medicare & Medicaid Services (2019)
Probability of death		0.73%	0.6%	0.9%	± 20%	0.073%	Beta	Tannenbaum et al. (2015)
(Breakdown)								
Non-medical		0	–	–	–	–	–	
Outpatient visit		0	–	–	–	–	–	
Non-fracture admission		6%	–	–	–	–	–	
Non-hip fracture admission		0.1%	–	–	–	–	–	
Hip fracture admission		20%	–	–	–	–	–	
MVC								
Hazard ratio	Lemborexant	2.2	1.64	2.95	95%CI	0.15	Log-normal	Same as zolpidem
	Suvorexant	2.2	1.64	2.95	95%CI	0.15	Log-normal	
	Zolpidem	2.2	1.64	2.95	95%CI	0.15	Log-normal	Hansen et al. (2015)
	Untreated insomnia	2.7	1.4	5.4	95%CI	0.34	Log-normal	Connor et al. (2002)
MVC rate (/year)	Younger	0.603%	0.482%	0.724%	± 20%	0.0603%	Beta	National Public Safety Commission and National Police Agency (2019)
	Older	0.483%	0.386%	0.580%	± 20%	0.0483%	Beta	
Probability of death		0.8%	0.77%	0.82%	95%CI	0.014%	Beta	
WPA								
Hazard ratio	Lemborexant	1.63	1.304	1.956	± 20%	0.163	Log-normal	Lombardi et al. (2010); Rosenberg et al. (2019)
	Suvorexant	1.63	1.304	1.956	± 20%	0.163	Log-normal	Lombardi et al. (2010); Rosenberg et al. (2019)
	Zolpidem	1.70	1.360	2.040	± 20%	0.170	Log-normal	Lombardi et al. (2010); Rosenberg et al. (2019)
	Untreated insomnia	1.79	1.22	2.62	95%CI	0.19	Log-normal	Lombardi et al. (2010)
WPA rate (/year)		1.02%	1.0195%	1.0282%	95%CI	0.0022%	Beta	Ministry of Health, Labour and Welfare (2019)
Probability of death		0.07%	0.060%	0.083%	95%CI	0.0059%	Beta	
Employment rate of those aged ⩾65 years		24.3%	–	–	–	0.0072%	Beta	Ministry of Internal Affairs and Communications (2018)
*Costs*								
Costs of drug (JPY/30 days)								
Lemborexant		2700	2160	3240	± 20%	–	–	NHI price (2020)
Suvorexant		3000	2400	3600	± 20%	–	–	
Zolpidem		900	720	1080	± 20%	–	–	
Costs for insomnia treatment								
initial medical cost (JPY/time)		2880	2304	3456	± 20%	288	Gamma	NHI medical fee (2020)
primary care visit (JPY/time)		730	584	876	± 20%	73	Gamma	
Additional primary care visits (per 30 days within time horizon)								
Lemborexant		1.3	–	–	–	0.0005	Gamma	claim database analysis
Suvorexant		1.3	–	–	–	0.0005	Gamma	
Zolpidem		1.3	–	–	–	0.0005	Gamma	
Untreated insomnia		0	–	–	–	–	–	Assumption
Severity of fallen cases and treatment								
Non-medical		44%	–	–	–	–	–	Niino et al. (2000)
Outpatient visit		48%	–	–	–	–	–	
Non-fracture admission		2.56%	–	–	–	–	–	Tannenbaum et al. (2015)
Non-hip fracture admission		2.56%	–	–	–	–	–	
Hip fracture admission		2.88%	–	–	–	–	–	
Costs for fall (JPY/event)		58 047	46 438	69 656	± 20%	5804.7	Gamma	
(Breakdown)								
Outpatient visit		3700	–	–	–	–	–	NHI medical fee (2020)
Non-fracture admission		188 280	–	–	–	–	–	NHI medical fee (2020)
Non-hip fracture admission		502 472	–	–	–	–	–	Oyama, Nakamura, Tsuchiya, Muto, and Yamamoto (2007)
Hip fracture admission		1 328 654	–	–	–	–	–	Kondo and Kawabuchi (2012)
Costs for MVC (JPY/event)		254 628	203 702	305 554	± 20%	25 462.8	Gamma	The General Insurance Association of Japan. Insurance data (2012)
Costs for WPA (JPY/event)		68 187	54 550	81 824	± 20%	6818.7	Gamma	Ministry of Health, Labour and Welfare (2019)
*Utilities*								
Baseline utility (in general population)								
Younger		0.928	0.925	0.930	95%CI	0.00104	Beta	Shiroiwa et al. (2016)
Older		0.847	0.844	0.850	95%CI	0.00174	Beta	Shiroiwa et al. (2016)
Utility decrement for no effect on chronic insomnia								
Base-case (sSOL)		0.026	0	0.053	95%CI	0.014	Beta	Ikeda et al. (2020)
Scenario (WASO)		0.005	–	–	–	–	–	Ikeda et al. (2020)
Scenario (LPS)		0.007	–	–	–	–	–	Ikeda et al. (2020)
Utility decrement for events								
Falls		0.036	0.029	0.043	± 20%	0.0036	Beta	Tannenbaum et al. (2015)
MVC		0.042	0.034	0.050	± 20%	0.0042	Beta	Spicer et al. (2011)
WPA		0	–	–	–	–	–	Assumption

SA, sensitivity analysis; PSA, probabilistic sensitivity analysis; s.e., standard error; CI, confidence interval; ER, extended release; sSOL, subjective sleep onset latency; WASO, wake after sleep onset; LPS, latency to persistent sleep; MVC, motor vehicle collisions; WPA, workplace accidents; JPY, Japanese yen; NHI, national health insurance.

#### Falls

The baseline risk of falls in good sleepers was obtained from the National Institute for Longevity Sciences, Longitudinal Study of Aging (NILS-LSA) in Japan (Niino et al., 2000). The risk of falls for untreated insomnia was derived from the general population of untreated patients with chronic insomnia from the Study of Osteoporotic Fractures (relative risk = 1.48) (Stone, Ensrud, & Ancoli-Israel, 2008). The risk of falls for lemborexant or suvorexant was derived from estimates of (1) the relative risk of falls for lemborexant and suvorexant compared with that of the general population for untreated patients (Stone et al., 2008) and (2) the NMA of the risk of falls for lemborexant-treated *v.* untreated patients [hazard ratio (HR) = 0.68] and *v.* suvorexant (HR = 0.85) (McElroy et al., 2021), as lemborexant and suvorexant are associated with a lower risk of falls than the risk in untreated insomnia patients. Thus, the HRs for the risk of falls for lemborexant and suvorexant compared with the risk in the general population were calculated as 1 ( = 0.68 × 1.48) and 1.26 ( = 0.85 × 1.48), respectively. An increased risk of falls with zolpidem IR compared with untreated persons was derived from a pooled analysis of 14 studies conducted in older adults (Treves, Perlman, Kolenberg Geron, Asaly, & Matok, 2018).

In a previous cost-effectiveness analysis from a US Medicare perspective, the model assumed that the number of extra falls per faller would be higher in a patient population that included a higher annual percentage of fallers (Tannenbaum et al., 2015). As data on the number of falls per year are available from the 2019 Medicare Current Beneficiary Survey (MCBS) (MCBS, 2019), this assumption could be verified by estimating the percentage of patients classified by variables that would differ in terms of the percentage of patients per year. Therefore, fallers in the 2019 MCBS were estimated to experience 1.957 [95% confidence interval (CI) 0.90–2.02] falls in a year (MCBS, 2019), and the number of extra falls per faller in the current model was estimated to be 0.957. The expected relationship between the annual number of falls per faller and the annual proportion of fallers was observed. Therefore, the estimated slope was determined from a regression analysis that was weighted by the precision of the estimated annual mean number of falls per faller [2.5 (95% CI 2.0–3.0)] and was used in the current model (e.g. the number of falls per capita in a treatment group with a 10% fall rate would be 0.25 higher than in a treatment group with a 20% fall rate).

The fatality rates following hospital admissions for falls (6% for no fracture, 0.1% for no hip fracture, and 20% for hip fracture) were obtained from the cost-effectiveness analysis from a US Medicare perspective (Tannenbaum et al., 2015). Falls not resulting in hospital admission were assumed to be minor injuries with no risk of death.

#### MVCs

The baseline risk and probability of death for MVCs in which MVC the insomnia patient was the driver were based on the incidence of collisions in Japan (NPA, 2019). The estimate of the increased risk of MVCs with zolpidem was sourced from an analysis of the US health plan for Washington state that included drivers aged >21 years (Hansen, Boudreau, Ebel, Grossman, & Sullivan, 2015). As there are no comparable data for suvorexant or lemborexant, the risk of these treatments was assumed to be the same as that of zolpidem.

The increased risk of MVCs in cases of untreated insomnia was sourced from a population-based case-control study (Connor et al., 2002). An adjusted OR of 2.7, determined from the comparison of those compared reporting five or more hours of sleep with those reporting five reported or fewer hours of sleep in the past 24 h, was used as the increased risk of MVCs associated with no treatment.

#### WPAs

The baseline risk and probability of death of WPAs were based on a survey conducted by the MHLW (MHLW, 2019). The increased risk of WPAs for untreated insomnia was obtained from a National Health Interview Survey conducted in the United States (Lombardi et al., 2010), which showed an increased risk of WPAs for workers who slept 5–5.9 h compared with that of workers who slept 7–7.9 h (OR 1.79). The total objective sleep duration at baseline in SUNRISE 1 was 327 min (OR 1.79) for treatment-unresponsive patients (Rosenberg et al., 2019); therefore, a weighted average value of 1.63 [0.207 × 1.0 +(1-0.207) × 1.79 = 1.63] was established based on the response rate of the drug treatment. As the employment rate of those aged ⩾65 years was estimated to be 24.3% (Ministry of Internal Affairs & Communications, 2018), the annual rate of injury for the older age group in the current model was assumed to be 24.3% of that of the younger group.

### Healthcare resource utilization and costs

All cost parameters are shown in Table 1. Information on patient visits to physicians was obtained from a health insurance claims database that comprises information on medical services, drug prescriptions, and diagnoses for company employees and their family members (JMDC Inc, 2020). Between March 2017 and August 2019, the frequencies of patient visits to physicians for the suvorexant and zolpidem IR groups were 1.33 and 1.29 per month, respectively. The mean value (1.3 visits per month) was used as the model input value for all treatments. There were no patient visits for untreated insomnia.

No treatment, outpatient treatment, and inpatient treatment were considered in the treatment of falls. Data from the NILS-LSA study showed that the treatment of fallers accounted for 56% of hospital visits and 8% of hospitalizations (Niino et al., 2000). According to the cost-effectiveness analysis from a US Medicare perspective, 32% of hospitalizations were for non-fractures, whereas 68% were for fractures; of the fracture admissions, 47% were for non-hip fractures, whereas 53% were for hip fractures (Tannenbaum et al., 2015). From these reports, the proportions of non-fracture hospitalizations, non-hip fractures, and hip fractures for all fallers were calculated to be 2.56, 2.56, and 2.88%, respectively. The cost of each treatment for a fall was determined from the literature or the national health insurance (NHI) cost that was applicable to the expected treatment in Japan.

MVC and WPA costs were obtained from general insurance data in Japan (The General Insurance Association of Japan, 2012), and treatment drug costs were obtained from the Japanese NHI drug prices for 2020. The daily drug costs for each treatment were JPY 92 for lemborexant, JPY 96.7, suvorexant, and JPY 28.4 for zolpidem IR, respectively. Zolpidem IR costs were calculated from the weighted average cost of the brand and available generics based on market share. The patients continued each of the drug treatment regimens throughout the analysis period. All medical costs were inflated to 2020 values.

### Utilities

The utilities are listed in Table 1. Utilities for responders were assumed to be the same as those for good sleepers whose utilities were equivalent to the values in the general population in Japan (Shiroiwa, 2016). According to the EuroQol 5-Dimensions (3-level version; EQ-5D-3L) data from SUNRISE 1, non-responders were assumed to experience utility decrement due to untreated insomnia (Ikeda et al., 2020). A total of 703 patients (responders: *n* = 116, non-responders: *n* = 587) who had sSOL responder rate data and who responded to the EQ-5D-3L questionnaire both at baseline and at one month were analyzed. The EQ-5D-3L data were scored based on tariffs from the UK (Szende, Oppe, & Devlin, 2007). A difference in the change from baseline QOL scores between treatment responders and non-responders was evaluated using an analysis of covariance, with age, sex, baseline QOL score, and comorbidities (depression and anxiety disorder) as covariates. The changes in utilities were 0.026 larger in the responder group than in the non-responder group (*p* = 0.068).

The disutility of falls was obtained from the cost-effectiveness analysis from a US Medicare perspective (Tannenbaum et al., 2015). As there was no utility decrement for a non-fracture hospital admission, this was conservatively assumed to be the same as for a fall.

The disutility associated with an MVC was the weighted average of the median utility loss for a person with an MVC in the United States in 2010 (Spicer, Miller, Hendrie, & Blincoe, 2011). No data were identified on the disutility for a WPA; therefore, conservatively, this value was set to zero. The disutility associated with the treatment was not considered because insufficient data were available.

### Sensitivity analysis

A scenario analysis was conducted for younger adults (18–64 years) and older adults (⩾65 years) using the parameters of the base-case analysis. Scenario analyses were also conducted using different indicators of the response rate. To assess the response rate, the scenario analyses were performed using a WASO of ⩽60 min, provided the mean baseline WASO was >60 min and was reduced by >10 min compared with baseline values, and an LPS ⩽20 min, provided the mean baseline LPS was >30 min. The disutility of insomnia was also applied to the values analyzed using the estimate derived from each specific response measure. The parameters are listed in Table 1.

Model parameter uncertainty was assessed via one-way analysis (OWA). The model inputs varied by 95% CIs where available, and by ± 20% of the base-case value when CIs were not publicly available. The probabilistic sensitivity analysis (PSA) was performed to assess the impact of individual parameter uncertainty on the model results. Random dispersion was specified using the reported standard error or an assumed standard error of 10% for cases in which it was not available (Table 1). The PSA followed a standard Monte Carlo approach consisting of 2000 randomly drawn simulations of the parameter values.

## Results

### Base-case analysis

In the base-case analysis, lemborexant was dominant over suvorexant and zolpidem IR, and was more cost-effective than untreated insomnia (Table 2). The mean estimated QALY was 0.4220 for lemborexant, 0.4204 for suvorexant, 0.4113 for zolpidem IR, and 0.4163 for untreated insomnia. The expected medical costs for lemborexant, suvorexant, and zolpidem IR were JPY 34 034 (USD 318), JPY 38 371 (USD 359), JPY 38 139 (USD 356), and JPY 15 383 (USD 144), respectively. The cost savings for lemborexant were JPY 4337 (USD 41) compared with suvorexant and JPY 4105 (USD 38) compared with zolpidem IR, while conferring incremental QALY benefits of 0.0016 and 0.0108, respectively. The ICER of lemborexant compared with that of untreated insomnia was JPY 3 220 975 (USD 30 103) /QALY.

**Table 2.** Analysis results

Strategy	Cost (JPY)	QALY	ICER: v. Suvorexant (JPY/QALY)	ICER: v. Zolpidem (JPY/QALY)	ICER: v. Untreated insomnia (JPY/QALY)
Base-case					
Lemborexant	34 034	0.4220	Dominant	Dominant	3 220 975
Suvorexant	38 371	0.4204	–	–	–
Zolpidem IR	38 139	0.4113	–	–	–
Untreated insomnia	15 383	0.4163	–	–	–
Scenario analysis (younger population)					
Lemborexant	33 169	0.4423	Dominant	Dominant	3 639 497
Suvorexant	37 108	0.4409	–	–	–
Zolpidem IR	34 941	0.4330	–	–	–
Untreated insomnia	13 819	0.4370	–	–	–
Scenario analysis (older population)					
Lemborexant	34 940	0.4008	Dominant	Dominant	2 849 652
Suvorexant	39 697	0.3989	–	–	–
Zolpidem IR	41 494	0.3885	–	–	–
Untreated insomnia	17 023	0.3945	–	–	–
Scenario analysis: WASO					
Lemborexant	34 034	0.4309	Dominant	Dominant	4 374 738
Suvorexant	38 371	0.4292	–	–	–
Zolpidem IR	38 139	0.4210	–	–	–
Untreated insomnia	15 383	0.4266	–	–	–
Scenario analysis: LPS					
Lemborexant	34 034	0.4301	Dominant	Dominant	4 198 480
Suvorexant	38 371	0.4284	–	–	–
Zolpidem IR	38 139	0.4199	–	–	–
Untreated insomnia	15 383	0.4256	–	–	–

JPY, Japanese yen; QALY, quality-adjusted life years; ICER, incremental cost-effectiveness ratio; WASO, wake after sleep onset; LPS, latency to persistent sleep; IR, immediate release

### Sensitivity analysis

For the analyses by age (younger adults, older adults), treatment with lemborexant was dominant compared with suvorexant or zolpidem IR, and it was cost-effective compared with untreated insomnia in both age groups. There were smaller incremental effects and cost savings with lemborexant *v.* the other therapies for the 18–64-year-old population compared with the ⩾65-year-old population.

In the scenario analysis with different indicators for the response rate, only QALY gains were affected because the response rate only impacted the utility decrement in the population with insomnia. The QALY gains with lemborexant, suvorexant, zolpidem IR treatment, and untreated insomnia were estimated to be 0.4309, 0.4292, 0.4210, and 0.4266, respectively, for the WASO response rates, and 0.4301, 0.4284, 0.4199, and 0.4256 for LPS, respectively. Lemborexant remained a dominant compared with suvorexant and zolpidem IR, and it was cost-effective compared with untreated insomnia in the analyses using all other scales for assessing treatment response (Table 2).

**Fig. 2.**
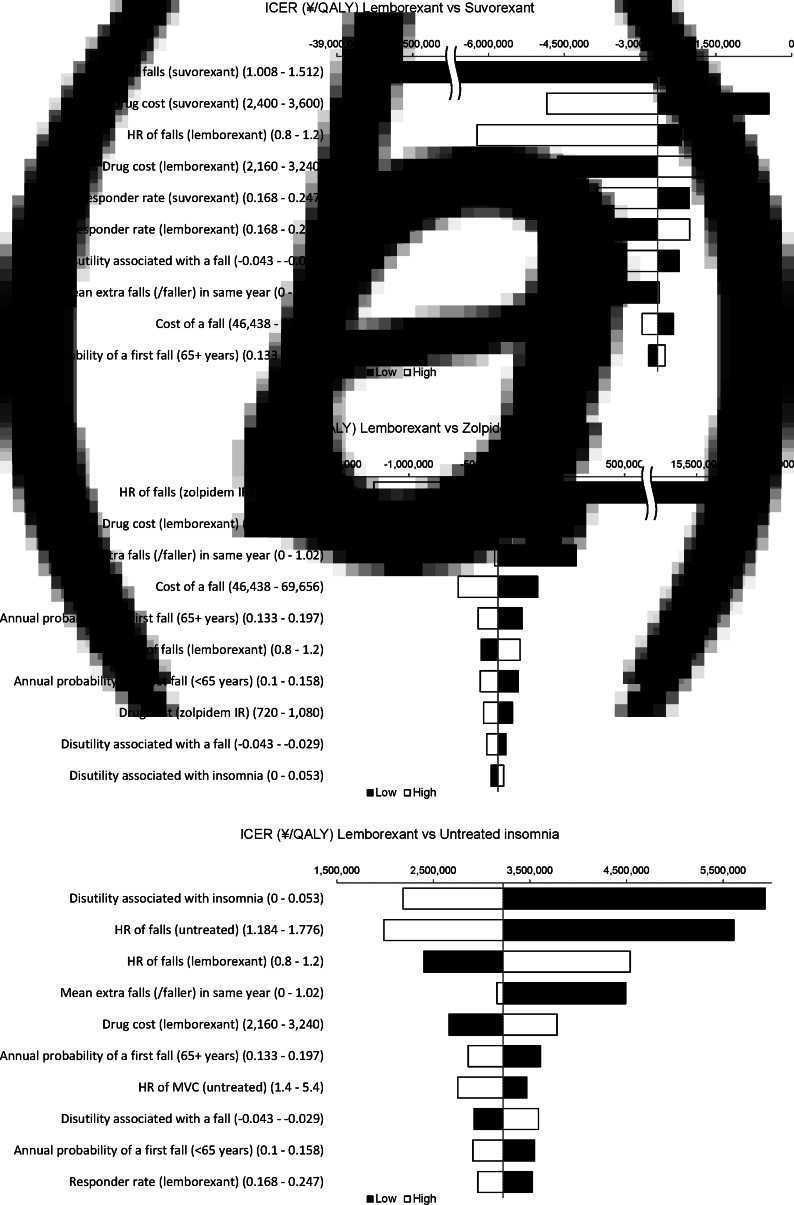
Tornado diagrams for comparisons of lemborexantversus suvorexant (a), lemborexant, *v.* zolpidem and (b), and lemborexant) *v.* untreated insomnia (c). HR, hazard ratio; ICER, incremental cost-effectiveness ratio; QALY, quality-adjusted life years.

The results of OWA are shown in Fig. 2. OWA of lemborexant *v.* suvorexant revealed that the model was most sensitive to variations in the risk (based on the HR) of a first fall with lemborexant. However, the results supported the predominance of lemborexant in all of the ranges examined. The OWA of lemborexant *v.* zolpidem IR revealed that the model was most sensitive to variations in the risk (based on the HR) of a first fall with zolpidem IR. When the HR for the first fall with zolpidem IR was below 2.03, the cost of lemborexant was greater than that of zolpidem IR; when the HR was below 1.11, the ICER for lemborexant exceeded JPY 5 million/QALY. The ICER was JPY 164 000/QALY when the average number of extra falls was zero (i.e. without accounting for re-falls). The analyses of lemborexant *v.* untreated insomnia revealed that the model was sensitive to variations in utility decrement for no treatment effect on chronic insomnia and the risk (based on the HR) of a first fall with untreated insomnia. When the utility decrement for no treatment effect on chronic insomnia was below 0.05 and the HR for a first fall with untreated insomnia was below 1.24, the ICER for lemborexant exceeded JPY 5 million/QALY.

**Fig. 3.**
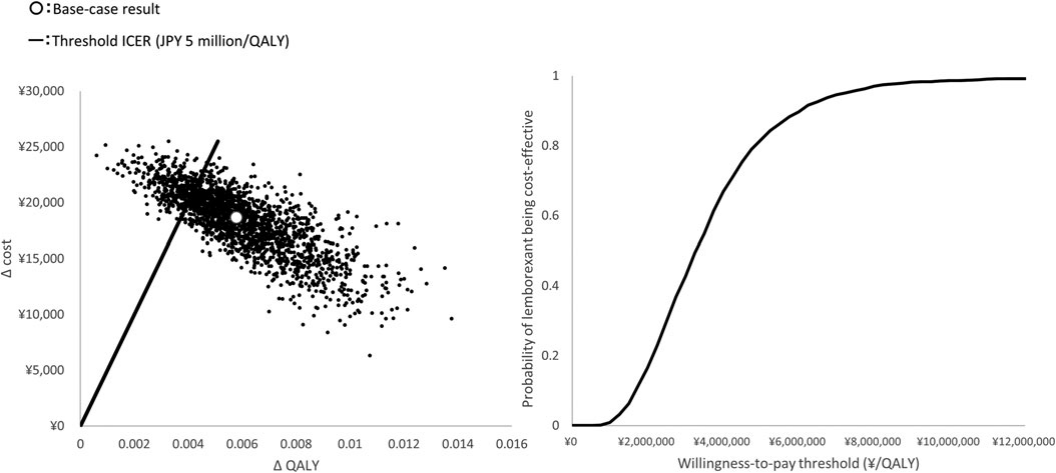
Results of the probabilistic sensitivity analysis (PSA) comparing lemborexant treatment with untreated insomnia. ICER, incremental cost-effectiveness ratio; QALY, quality-adjusted life years; WTP, willingness to pay.

The PSA results showed that lemborexant was cost-saving compared with suvorexant or zolpidem IR in 90.7 and 62.0% of the simulations, and lemborexant was cost-effective compared with suvorexant and zolpidem IR in 95.7 and 93.8% of the simulations that used a WTP threshold of JPY 5 million/QALY. In comparison with untreated insomnia, lemborexant would be cost-effective with an 81.7% probability (Fig. 3).

## Discussion

The present study evaluated the cost-effectiveness of lemborexant, compared with suvorexant, zolpidem IR, and no treatment, in Japan. In the base-case analysis, lemborexant was less expensive, provided QALY benefits over the active comparators, and was cost-effective compared with untreated insomnia. The sensitivity analyses supported these results.

The incremental effect was lower in the scenario analysis because the probability of a fall was lower in younger people. However, the result did not change the trend of the base-case results. The results from the other response rate indicators, such as the LPS or WASO measures, supported the dominance of lemborexant, suggesting limited effects of the choice of scale for the responder definitions in our analysis. Since this analysis was conducted from the healthcare perspective typically adopted by payers in Japan and only assessed direct medical costs, it did not consider other costs that are likely to have a major impact on cost-effectiveness. These costs include, for example, indirect costs associated with absenteeism and work productivity losses in the economically active population, costs for carers, modifications to the home to minimize the risk of a subsequent fall, costs associated with motor vehicle repairs, and costs to repair damage in the workplace because of accidents.

The incidence of insomnia, characterized by difficulties associated with sleep onset, maintenance, or both, increases with age (Kim, Uchiyama, Okawa, Liu, & Ogihara, 2000). The Japanese guidelines published in 2014 (Mishima, 2015) recommend using non-BZDs for treating primary insomnia in the elderly because of the low risk of adverse events (AEs) compared with those that occur with BZDs based on the available evidence of the time. Because non-BZDs have high selectivity for the *ω*1 subunit of gamma-aminobutyric acid (GABA) receptors, they are thought to exert a smaller effect on muscle relaxation and be safer than BZDs (Uchiyama, 2019). Despite these assumptions, patients administered non-BZDs still experience unsteadiness and have an increased risk of fractures due to falls (Treves et al., 2018). Additionally, BZDs and non-BZDs are not necessarily efficacious for treating sleep maintenance difficulties (Rosenberg, 2006). Lemborexant may serve as a future treatment option for insomnia because, in addition to improving both sleep onset and maintenance, it does not mediate the activity of GABA receptors, resulting in less unsteadiness and less muscle relaxation, which may reduce the risk of fall fractures (Uchiyama, 2019).

There might be several confounding factors that could change the cost-effectiveness results of using one DORA over another. These need to be discussed as insomnia is often associated with other mental and physical disorders and is more common in older people. Regarding age, DORA appears to be less likely to cause adverse events than other classes of hypnotics in the elderly. A population pharmacokinetics (PK) model based on clinical reported a low degree of association with lemborexant treatment-emergent adverse events (TEAEs) and exposure in adults and the elderly with chronic insomnia. Therefore, lemborexant can be safely administered without dose adjustment for age (Lalovic et al., 2020). It has been reported that there is no difference in the incidence of adverse events with age when suvorexant is used at the prescribed dosage (20 mg for adults and 15 mg for the elderly per day) (Herring et al., 2016). Postural stability may be associated with the risk of falls. However, there was no significant difference in body sway reported for lemborexant (5 or 10 mg) compared with placebo in the morning waketime in healthy adults aged 55 years or older, while zolpidem-ER did show a significant difference (worsening) compared with placebo (Murphy, Kumar, Zammit, Rosenberg, & Moline, 2020). It was reported that suvorexant (30 mg) also has no difference in postural stability compared with PBO or zolpidem (5 mg) 8 h after dose (Bland et al., 2021). There were no significant differences in efficacy or safety of lemborexant treatments between patients with and without comorbidities, i.e., history of depression, anxiety, gastro-esophageal reflux disease, migraine, hypertension, diabetes, and renal dysfunction [Nierenberg et al., 2020; ‘Pharmaceuticals and Medical Devices Agency (PMDA) Shinsa Houkokusho (Review Report),’, 2020]. Conversely, suvorexant was used for patients with insomnia without comorbidities in the phase-3 studies along with DSM-IV; therefore, no such data on the efficacy or safety of suvorexant in insomnia patients with comorbidities are reported. In terms of cognitive function, in patients with mild to moderate Alzheimer's disease who also had sleep disturbances, neither treatment with lemborexant nor suvorexant adversely affected cognitive function as assessed by the MMSE (Herring et al., 2020; Moline et al., 2021). In patients with obstructive sleep apnea (OSA), there were no clinically important respiratory effects during sleep in patients with mild OSA in patients between lemborexant 10 mg *v.* placebo (Cheng et al., 2020) and in patients with mild to moderate OSA between suvorexant 40 mg *v.* placebo (Sun et al., 2016). Based on the above discussion, confounding factors such as age, cognitive function, postural stability, and other co-morbidities were examined. At least from the reports currently available, those factors do not appear to significantly affect the efficacy or safety of lemborexant and suvorexant; therefore, the impact on cost-effectiveness as we analyzed is limited. However, most insomnia patients in clinical practice suffer from co-morbidities, and there is a paucity of reports on their treatment effects on insomnia. Therefore, it should be noted that the results of this study are based on an analysis of a limited amount of information.

### Limitations

Although the uncertainty of the model parameters was assessed by PSA, which showed robustness, several limitations must be considered when assessing the validity of this model. Firstly, the response rate measured at one month in SUNRISE 1 was assumed to last six months. Although the 6-month-long SUNRISE 2 (Kärppä et al., 2020; Yardley et al., 2020) study was conducted to examine the long-term efficacy and tolerability of lemborexant compared with that of placebo, the results of SUNRISE 1 were used because SUNRISE 2 did not include a direct comparison with zolpidem ER or any other treatments. However, this assumption was reasonable because significant benefits over placebo were observed at the end of the six months in SUNRISE 2 and at most time points assessed over the entire 6-month period, indicating long-term, sustained efficacy of lemborexant (Kärppä et al., 2020). Secondly, no studies have directly compared suvorexant with lemborexant; the sSOL response rate used to measure efficacy was conservatively assumed to be the same for both drugs. The results of an NMA showed a statistically significant improvement in sSOL assessments after three months of treatment with lemborexant compared with that of suvorexant (McElroy et al., 2021). In addition, Kishi et al. (2020) recently reported that both 5 and 10 mg lemborexant outperformed suvorexant (20/15 mg) and zolpidem ER (6.25 mg) in terms of the subjective time to sleep onset after one week. However, the present study conservatively assumed equivalent efficacy despite the potential for lemborexant to be better than suvorexant. The advantage of lemborexant over suvorexant was, thus, potentially underestimated in this cost-effectiveness model. Thirdly, the disutility associated with treatment was not considered because there were insufficient data to calculate the magnitude of disutility. In SUNRISE 1, headache, somnolence, urinary tract infections, nasopharyngitis, upper respiratory tract infections, infections, and dizziness were reported as treatment-emergent AEs (Rosenberg et al., 2019); however, they were not considered in the current analysis because the overall incidence of treatment-emergent AEs was similar between the treatment groups and did not have a significant impact on either QOL or cost. In addition, although the potential for rebound insomnia and withdrawal symptoms may raise concerns about anti-insomnia drugs, no such effects were reported with lemborexant in either SUNRISE 1 or SUNRISE 2 (Kärppä et al., 2020; Rosenberg et al., 2019; Yardley et al., 2020). Fourthly, this analysis was conducted solely to evaluate the impact and costs of using lemborexant in the Japanese population, and healthcare systems, costs, and treatment guidelines could vary in other markets. Finally, the treatment effects in the analysis were based on the clinical trial; however, various patients with characteristics influencing the treatment effects will use these drugs in actual clinical practice. It is expected that further verification of the cost-effectiveness of lemborexant based on the treatment effect collected in actual clinical practice in the future.

## Conclusions

According to the findings of this cost-effectiveness analysis of patients with insomnia from the Japanese payer's perspective, lemborexant was dominant over suvorexant and zolpidem IR. It was cost-effective compared with untreated insomnia. Although generalizability to real-world practice should be carefully considered, lemborexant may represent a better investment for treating insomnia in the healthcare system in Japan.
