# Cost-effectiveness analysis of lemborexant for treating insomnia in Japan: A model-based projection, incorporating the risk of falls, motor vehicle collisions, and workplace accidents – CORRIGENDUM

**DOI:** 10.1017/S0033291722001854

**Published:** 2022-10

**Authors:** Shunya Ikeda, Mie Kasai Azuma, Kenichi Fujimoto, Hidetoshi Shibahara, Sachie Inoue, Margaret Moline, Mika Ishii, Kazuo Mishima

**Affiliations:** 1 Department of Public Health, School of Medicine, International University of Health and Welfare, Narita, Japan; 2 Eisai Co., Ltd., Tokyo, Japan; 3 CRECON Medical Assessment Inc., Tokyo, Japan; 4 Graduate School of Health and Welfare, International University of Health and Welfare, Tokyo, Japan; 5 Eisai Inc., Woodcliff Lake, NJ, USA; 6 Department of Neuropsychiatry, Akita University Graduate School of Medicine, Akita, Japan

This article was published in Psychological Medicine with the reference ‘Ikeda, S., Azuma, M., Fujimoto, K., Shibahara, H., Inoue, S., Moline, M., & Ishii, M. (2020). PMH8 EQ-5D Analysis in Patients with Insomnia: Change of Quality of Life in Lemborexant Phase 3 Trial Sunrise 1. Value in Health, 23. doi:10.1016/j.jval.2020.08.1086’ missing. The online version of this article has been updated to include this reference.

The authors apologise for this error.
